# Characterization of the intestinal microbiota during *Citrobacter rodentium* infection in a mouse model of infection-triggered Parkinson’s disease

**DOI:** 10.1080/19490976.2020.1830694

**Published:** 2020-10-16

**Authors:** Tyler Cannon, Anshul Sinha, Louis-Eric Trudeau, Corinne F. Maurice, Samantha Gruenheid

**Affiliations:** a Department of Microbiology and Immunology, McGill University, Montreal, QC, Canada; b Department of Pharmacology and Physiology, Department of Neuroscience, GRSNC, Université de Montréal, Montreal, QC, Canada

**Keywords:** Gut microbiota, Parkinson’s disease, infection, gut-brain axis, autoimmunity

## Abstract

Parkinson’s disease (PD) is a neurodegenerative disorder that has been shown to be influenced by the intestinal milieu. The gut microbiota is altered in PD patients, and murine studies have begun suggesting a causative role for the gut microbiota in progression of PD. We have previously shown that repeated infection with the intestinal murine pathogen *Citrobacter rodentium* resulted in the development of PD-like pathology in *Pink1*^−/-^ mice compared to wild-type littermates. This addendum aims to expand this work by characterizing the gut microbiota during *C. rodentium* infection in our *Pink1*^−/-^ PD model. We observed little disturbance to the fecal microbiota diversity both between infection timepoints and between *Pink1*^−/-^ and wild-type control littermates. However, the level of short-chain fatty acids appeared to be altered over the course of infection with butyric acid significantly increasing in *Pink1*^−/-^ mice and isobutyric acid increasing in wild-type mice.

## Introduction

Parkinson’s disease (PD) is the second most common neurodegenerative disease in the world, affecting more than 10 million people worldwide.^1,2^ PD is associated with a range of symptoms including movement deficits caused by the destruction of dopaminergic neurons. In humans, motor symptoms manifest as tremors, slowness of movement (bradykinesia), muscle rigidity, and altered gait and balance.^3-5^ The molecular mechanisms underlying the death of the dopaminergic neurons in PD remain poorly understood.^1,6,7^ Although the majority of PD cases (>85%) are idiopathic in nature with no known cause, familial forms of the disease are thought to be strongly linked to inheritance of mutations within one of many PD-associated causative genes including *PINK1*.^8^ Inheritance of biallelic *PINK1* mutations is associated with early-onset PD.^8,9^

The PINK1 protein is largely known for its role in mitochondrial quality control.^10,11^ PINK1 is a kinase that is stabilized on the outer mitochondrial membrane when the mitochondrion loses its membrane potential, initiating a pathway resulting in mitophagy.^12^ The lack of functional PINK1 in familial forms of PD was thus thought to promote the accumulation of dysfunctional mitochondria causing dopaminergic neuronal death; however, this has proven difficult to validate. Notably, *Pink1*^−/-^ mice, although having dysfunctional mitochondria, fail to experience neurodegeneration or significant motor impairment.^13^ An alternative function for PINK1 was recently discovered whereby it suppresses the presentation of mitochondrial peptides on MHC I to the immune system in response to bacterial lipopolysaccharide (LPS),^14^ suggesting that PINK1’s role in PD may be immunological and influenced by external factors.

Indeed, despite the strong association between *PINK1* mutations and PD, recent epidemiological evidence has noted that the manifestation of familial forms of PD may not be reliant solely on genetics but can also be influenced by external factors.^15,16^ A growing body of research has begun to reveal the importance of peripheral organs like the gut in PD. At least five independent studies have now demonstrated that patients with inflammatory bowel diseases (IBD) have an increased PD incidence rate.^17-21^ Further, treatment for IBD with anti-TNF therapy has been documented in one study to reduce PD incidence rates in IBD patients by over 75%.^20^ A recent report also correlated appendectomies to decreased PD incidence rates.^22^ In animal studies, chronic stress-induced intestinal dysfunction was shown to correlate with exacerbated PD-like symptoms in a rotenone-induced mouse model of PD.^23^ Considerable research on the intestinal microbiota has also been carried out in both humans and animal models to examine whether a PD-specific microbiota exists and whether it is a causative factor in PD progression (reviewed in Cryan et al.^24^ Sun et al.,^25^ Tremlett et al.^26^). It is still unclear how the gut microbiota may affect brain physiology, although a number of studies have now highlighted how an altered microbiota can lead to enhanced TLR4 signalling that results in changes in glial cell function,^27,28^ while others have noted how the microbiota can affect blood brain barrier permeability.^29^ Notably, a human PD-patient derived gut microbiota was also shown to exacerbate PD-like pathology in an ⍺-synuclein overexpressing mouse model of PD, and this was proposed to be mediated by the altered production of short-chain fatty acids (SCFAs).^30^

Given this evidence, intestinal inflammation a plausible mechanism by which the gut-brain axis may be aggravated. Our recently published work sought to test the hypothesis that inflammatory signaling in the intestine could influence the development of PD phenotypes in *Pink1*^−/-^ mice.^31^ To do so, we used *Citrobacter rodentium*, a murine intestinal pathogen modeling pathogenic *Escherichia coli* infections of humans. In this work, we showed that *C. rodentium* infection of *Pink1^−/-^* mice, but not their WT littermates, induced the presentation of mitochondrial antigens on MHC I and the subsequent formation of anti-mitochondrial CD8+ T cells. Further analysis revealed that these anti-mitochondrial CD8+ T cells were detectable within the central nervous system. Following three additional exposures to *C. rodentium* over four months, *Pink1*^−/-^ mice displayed reduced spontaneous locomotion in an open field chamber and motor deficits as measured by decreased ability to descend a pole, a deficit that was reversed by treatment with _L_-DOPA, a dopamine synthesis precursor. Together, this data led us to hypothesize a two-hit model proposing that the onset of PD-like pathology is dependent on both an external factor (intestinal infection) and a genetic component (the *Pink1*^−/-^ background).

The model put forward in our previous paper is that anti-mitochondrial CD8 T cells induced by infection in the *Pink1*^−/-^ mice enter the CNS, causing damage to dopaminergic neurons and leading to motor symptoms. However, this does not rule out the possibility that other features of *C. rodentium* infection may also contribute to the development of PD-like symptoms in *Pink1^−/-^* mice. *C. rodentium* infection induces temporal shifts in the mouse intestinal microbiota including an expansion of *Enterobacteriaceae*.^32,33^ As described above, gut microbiota alterations, such as an expansion in *Enterobacteriaceae* and *Verrucomicrobia*, have been implicated in PD.^25^ This raises the possibility that microbiota differences between wild-type (WT) and *Pink1*^−/-^ mice, either before, during, or after infection could be implicated in the infection-induced PD-like phenotypes observed. To gain insight into the role of the microbiota in our model, this addendum aims to characterize changes in the gut bacterial microbiota and SCFA production during *C. rodentium* infection and compare these changes between *Pink1*^−/-^ mice and their WT littermates.

## Gut bacterial diversity

Susceptibility to *C. rodentium* varies depending on genetic background, with resistant C57BL/6 and 129S1 mouse lines experiencing only mild self-limiting colitis compared to the severe colitis, diarrhea, and weight loss observed in susceptible backgrounds.^34-36^ Infection kinetics of *C. rodentium* also vary between mouse backgrounds, although the loads generally peak between 9 and 13 days post infection (PI) with a fecal burden reaching upwards of 10^9^ colony forming units per gram of feces.^37^ In our previous study, we used WT and *Pink1*^−/-^ littermate mice that had been previously generated and used for PD studies on a B6.129 mixed background.^31^ Both WT and *Pink1*^−/-^ mice presented with similar and mild self-limiting colitis. We also reported that both WT and *Pink1*^−/-^ mice become colonized to a similar degree, with the infection peaking at day 13 PI with between 10^8^ and 10^9^ fecal colony forming units per gram of feces. The infection was completely cleared in all mice by day 26 PI. This kinetic allowed us to compare the influence of *C. rodentium* infection on the microbiota by collecting fecal samples prior to infection (pre), at day 13 (peak), and at day 26 (post) for 16S rRNA gene sequencing. To do this, we used the Illumina MiSeq sequencing platform and amplified the V4 region of the gene using the 515F and 806R primers. The sequencing data was analyzed using QIIME2 version 2019.7.^38^

**Figure 1.**
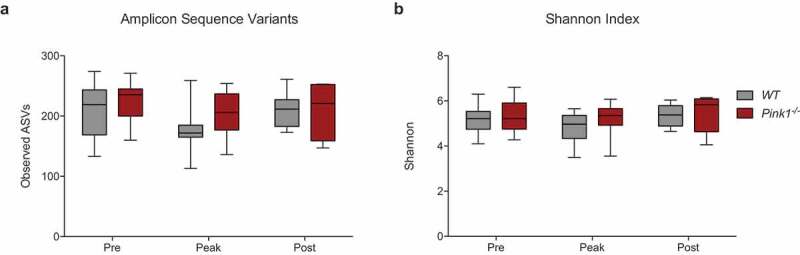
**The α¯diversity within the intestinal microbiota remains steady and comparable between WT and *Pink1*^−/-^ mice during *Citrobacter rodentium* infection**. (a) Comparison of the number of unique amplicon sequence variants observed upon rarefaction of the sequencing results to a depth of 20,000 reads. (b) Comparison of the Shannon index. Statistics determined via pairwise Kruskal-Wallis test. (WT: Pre N=16, Peak N = 15, Post N = 6. *Pink1*-/-: Pre N = 18, Peak N = 16, Post N = 5). Data represented as quartile box and whisker blots.

To determine how the *Pink1*^−/-^ genetic background might affect fecal microbial diversity during *C. rodentium* infection, we rarefied our sequences to a depth of 20,000 reads and compared the number of unique amplicon sequencing variants (ASVs) across time points and genotypes. The number of ASVs in both WT and *Pink1*^−/-^ mice largely remained stable and comparable throughout the infection (Figure 1(a)). Although not significant, there was a trend for the number of ASVs to decrease at the peak of infection in both genotypes. To measure microbial diversity between time points and genotypes, we next compared the Shannon index. Consistent with the number of ASVs, the Shannon index also remained largely unchanged, with a small, non-significant decrease at the peak of infection (Figure 1(b)).

**Figure 2.**
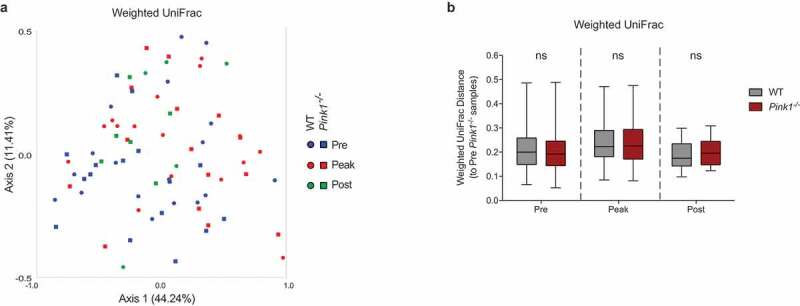
**The gut microbiota bacterial communities are similar between WT and *Pink1*^−/-^ mice during *C. rodentium* infection**. (a) Principle coordinate analysis between timepoints during the course of *C. rodentium* infection within WT mice and within *Pink1*^−/-^ mice, determined using the weighted UniFrac diversity metric. (b) Graphical analysis of the weighted UniFrac diversity metric. Statistics determined via PERMANOVA analysis, ns = not significant. Data represented as quartile box and whisker blots.

We next sought to compare the bacterial communities between genotypes and time points by assessing β-diversity. To do so, we generated principle coordinate analysis (PCoA) plots using weighted UniFrac. The weighted UniFrac PCoA plot, which considers relative abundance and evolutionary relatedness, revealed no distinct clustering based on genotype or timepoint (Figure 2(a)). This was also confirmed by PERMANOVA analysis (Figure 2(b)). Combined with the data highlighting the number of ASVs and the Shannon index, our results suggest that *C. rodentium* infection does not significantly impact the diversity of the fecal microbiota in our model system over the course of infection. Further, the infection does not appear to interact with the *Pink1*^−/-^ genotype to result in a distinct fecal microbial community. These results are compatible with other studies that have suggested that *C. rodentium* does not greatly disturb the bacterial communities present within the intestinal lumen or feces of mice^32,33^

## Taxonomic analysis of the gut bacterial microbiota

The composition of the murine intestinal microbiota, which is primarily comprised of species from the phyla *Bacteroidetes* and *Firmicutes*, can influence both *C. rodentium* colonization rates and infection susceptibility.^39,40^ Studies have now highlighted that ablation of the gut microbiota via antibiotic treatment can increase susceptibility to *C. rodentium*.^36,39^ Further, transfer of the microbiota from a resistant mouse strain can confer resistance to a susceptible mouse strain.^41^ Both an increased diversity within the phylum *Firmicutes* and a higher ratio of *Bacteroidetes* to *Firmicutes* have been associated with decreased susceptibility to *C. rodentium*.^42^ Segmented filamentous bacteria have also been shown to inhibit *C. rodentium* colonization at the mucosal surface, likely through immune mechanisms.^40^

Conversely, *C. rodentium* is also able to alter the intestinal environment by causing an expansion of undifferentiated epithelial cells, altering the expression profile of host cells to favour oxidative phosphorylation, and increasing oxygen levels at the mucosal surface.^32,43^ Notably, this can result in decreased *Bacteroidetes* and *Firmicutes* and a significant expansion of *Enterobacteriaceae*.^32^ At the peak of infection, *C. rodentium* comprises approximately 10% of luminal bacteria and between 40-90% of mucosal bacteria.^32,33^ It has been shown that as a result of PINK1’s role in mitochondrial maintenance, cells in *Pink1*^−/-^ mice experience a deficit of ATP production, loss of mitochondrial membrane potential, and overall lower mitochondrial respiratory potential.^13^ Thus, we considered the possibility that, during *C. rodentium* infection, *Pink1*^−/-^ mice might have decreased mucosal oxygen levels that would subsequently lead to alterations in microbiota compared to WT mice.

**Figure 3.**
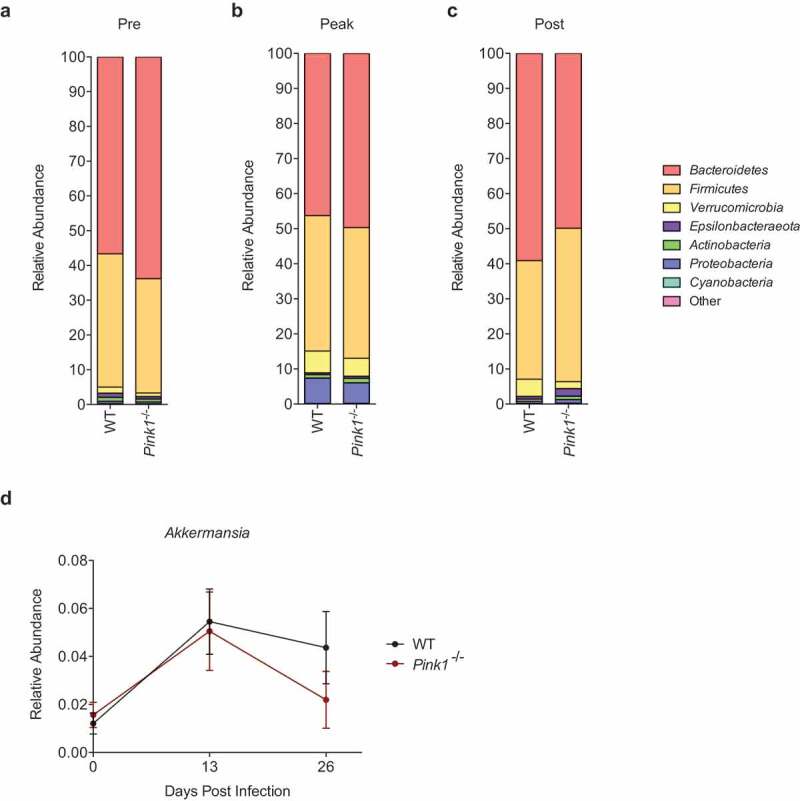
**Taxonomic analysis and comparison of the intestinal bacterial microbiota at the phylum and genus level during *Citrobacter rodentium* infection between WT and *Pink1*^−/-^ mice**. (a) Average relative abundance of major phyla present in WT and *Pink1*^−/-^ mice (a) pre-, (b) peak-, and (c) post-infection with *C. rodentium*. (d) Relative abundance of the genus *Akkermansia* over the course of *C. rodentium* infection. Data represented as mean +/- SEM.

To determine the taxonomic composition of the microbiota within our sample groups, we generated bar plots at the phylum level and compared between time points and genotypes. In accordance with previous reports,^32,33^ the major phyla present in both the WT and *Pink1*^−/-^ mice at steady state were *Bacteroidetes* and *Firmicutes* (Figure 3(a)). We also observed the presence of *Verrucomicrobia, Epsilonbacteraeota, Proteobacteria, Cyanobacteria*, and *Actinobacteria* at low proportions. Prior to infection, no significant differences in composition were observable between WT and *Pink1*^−/-^ mice at the genus level. This was further confirmed using an Analysis of the Composition of the Microbiota (ANCOM), a statistical test which determined no significance between genotypes at any time point. Notably, the ratio of *Bacteroidetes* to *Firmicutes* in our mice was high, consistent with the resistant phenotype we observed.^31^ No segmented filamentous bacteria were detected. At the peak of infection, we observed an expansion in the phylum *Proteobacteria*, which increased to approximately 6-10% in both WT and *Pink1*^−/-^ mice, which is likely a direct reflection of the increase in *C. rodentium* loads during infection (Figure 3(b)). The ANCOM analysis showed no significant differences between genotypes at this time point, providing further evidence that the *C. rodentium* colonization levels in WT and *Pink1*^−/-^ mice are comparable, as we have previously reported.^31^ The relative percent abundance of *C. rodentium* also corroborates previous 16S analyses of luminal contents.^32^ Post infection, we observed that the relative percent abundance of *Proteobacteria* reverted to pre-infection levels in both genotypes, likely reflecting that the infection has been cleared by this time point (Figure 3(c)).

We also noted a modest increase in the phylum *Verrucomicrobia* in both WT and *Pink1*^−/-^ mice at the peak of infection and, although not significant (Figure 3(c)), WT mice demonstrated a trend towards an elevated percentage of *Verrucomicrobia* compared to *Pink1*^−/-^ mice post infection (Figure 3(d)). It is interesting to note that *Verrucomicrobia*, which is increased at the peak of infection, is largely dominated by the genus *Akkermansia. Akkermansia muciniphila* is a Gram-negative mucin-degrading microbe associated with anti-inflammatory immune signatures in humans.^44^ It has been reported to be a signature in PD patient fecal microbiotas compared to healthy controls,^45,46^ an observation that we were not able to model in our mice. A higher proportion of *Akkermansia*, a similar finding to our own, has however been observed in a chronic stress-induced gut dysfunction mouse model of PD,^23^ suggesting this difference might be consistent in mouse models. Overall, the lack of significant compositional changes between genotypes at any time point combined with the diversity metrics suggests that the fecal microbiota of WT and *Pink1*^−/-^ mice remains largely comparable throughout infection with *C. rodentium*.

## Short-chain fatty acid analysis

A growing body of evidence has highlighted the potential implication of bacterially generated short-chain fatty acids (SCFAs) in PD. In humans, decreased levels of the fecal SCFAs butyrate, propionate, and acetate have been observed in PD patient fecal samples compared to matched controls.^47^ In a chemically induced mouse model of PD, a fecal microbiota transfer leading to increased propionic and isobutyric acid levels correlated with increased dopamine levels.^48^ Conversely, a recent study from Sampson et al. demonstrated that transferring a human PD-derived microbiota into a mouse alpha-synuclein overexpression PD model resulted in increased butyric acid and propionic acid levels, followed by an increase in PD-like symptoms, and administration of a mix of SCFAs into germ-free mice recapitulated some of the observed effects.^30^

SCFAs are highly implicated in intestinal barrier integrity and immunological responses. Butyric acid functions as a histone deacetylase inhibitor and is instrumental in maintenance of intestinal barrier integrity.^49,50^ Notably, a deficit in butyric acid has been associated with intestinal disorders in humans.^51,52^ Changes in SCFAs within our model system might therefore provide a plausible mechanism by which intestinal permeability is altered, allowing bacteria and/or bacterial molecules such as LPS to initiate and exacerbate pathological immune responses in the lamina propria. Indeed, we have also previously shown that LPS is likely a key player in the induction of mitochondrial antigen presentation. Additionally, butyric acid promotes the differentiation and genesis of regulatory T cells by increasing the acetylation of the *foxp3* locus,^53^ and acetate has been shown to increase the suppressive potential of regulatory T cells^54^ providing another possible mechanism by which differences in SCFAs between WT and *Pink1*^−/-^ mice might impact the host response to *Citrobacter* infection.

**Figure 4.**
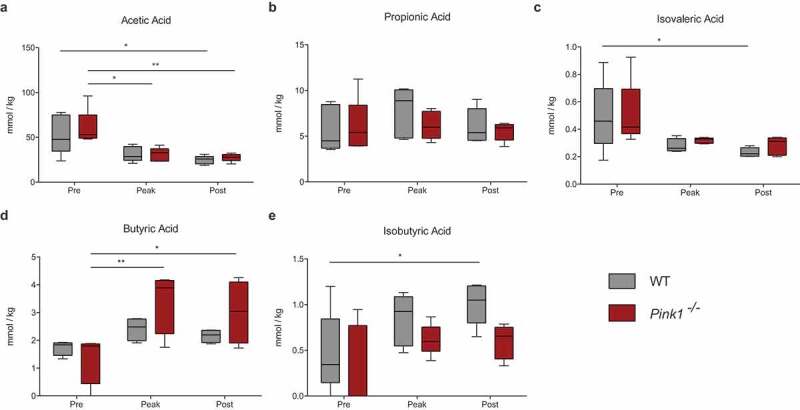
**Comparison of fecal short-chain fatty acid concentrations between WT and *Pink1*^−/-^ mice during *Citrobacter rodentium* infection**. Analysis of (a) acetic acid, (b) propionic acid, (c) isovaleric acid, (d) butyric acid, and (e) isobutyric acid fecal concentrations via gas chromatography. *P<0.05, **P<0.01 Significance determined via two-way ANOVA with a Tukey post-hoc test. (N = 5 per group). Data represented as quartile box and whisker plots.

To determine the levels of various SCFAs, we analyzed fecal samples from the WT and *Pink1*^−/-^ mice before, during, and after infection with *C. rodentium* via gas chromatography (Microbiome Insights). Acetic acid levels were comparable between genotypes at all time points, but significantly decreased at the peak of infection and remained low post infection (Figure 4(a)). Propionic acid levels remained steady and comparable at all time points (Figure 4(b)). There was a trend for the level of isovaleric acid to decrease with infection, but it remained low and comparable between genotypes at all time points (Figure 4(c)). Notably, butyric acid levels significantly increased at the peak of infection selectively in *Pink1*^−/-^ mice and remained high post infection (Figure 4(d)). Contrarily, isobutyric acid significantly increased with infection only in WT mice (Figure 4(e)). Valeric acid and hexanoic acid were below the limit of detection for all sample groups (data not shown).

The increased level of butyric acid in infected *Pink1*^−/-^ mice (Figure 4(d)) is notable in light of the results observed in the study by Sampson et al. in which SCFA treatments alone were sufficient to induce some PD-like symptoms in germ-free α-synuclein over-expressing mice.^30^ Both results remain contradictory to human studies where butyric acid levels are consistently observed to be lower in PD patients – it is still unclear how to consolidate this data. It is possible that the elevated levels of butyric acid observed in mouse studies reflect early time points in PD pathology, while lower butyric acid levels observed in humans reflect end points.

Based on the effects of butyric acid’s actions as a histone deacetylase inhibitor in enterocytes, higher butyric acid levels would be predicted to improve barrier function rather than induce intestinal dysfunction or aggravate the gut-brain axis. Further, butyric acid’s effects on regulatory T cells^53^ would be predicted to dampen rather than exacerbate immune pathology. An increase in butyric acid in response to infection in *Pink1*^−/-^ mice could therefore potentially represent a compensatory response to protect intestinal integrity. Rectal administration of butyrate also ameliorates *C. rodentium* induced cell injury,^55^ suggesting that the increased level of butyrate we observe can represent a protective response in our model. In our previous work, we showed that *C. rodentium* infection in *Pink1*^−/-^ mice leads to the production of autoreactive CD8+ T cells and an increased expression of the pro-inflammatory marker IFNγ^³¹^. While CD4+ T cells were not analyzed in this paper directly, no evidence was suggestive of increased immune regulation via regulatory T cells; however, a more thorough study would be needed to confirm this. Little is known about the functions of isovaleric and isobutyric acid; both were observed at similar levels between PD patients and healthy matched controls.^47^

One important caveat of the studies described here is that the microbiome and SCFA analyses were performed during the course of a single *C. rodentium* infection. Our original study demonstrated that anti-mitochondrial CD8+ T cells were generated at the peak of a single infection. However, the mice that developed motor symptoms had been exposed to *C. rodentium* four times over the course of four consecutive months. Since *C. rodentium* only colonized the mice to detectable levels during the first infection, suggesting that *C. rodentium*-induced changes to the intestinal environment are likely to occur during the first infection, we chose to focus our analysis within this time frame. It remains to be determined whether subsequent exposures to *C. rodentium* might lead to other changes in microbiome or SCFA composition between WT and *Pink1*^−/-^ mice.

## Concluding remarks

Currently the literature regarding the relationship between the intestinal microbiota and PD remains largely correlative in humans. The complex nature of PD, in that there are multiple suspected pathological mechanisms in idiopathic and familial forms, further adds to the complexity of studying the role of the gut and the microbiota within a population. The current murine model systems that exist for PD also tend to be limited to specific features of the disease and generally fail to encompass the multiple aspects and symptoms of PD. Nevertheless, regulation of the immune system via the intestinal milieu provides a plausible mechanism by which the gut-brain axis might drive PD.

Here we looked at how the host-microbe interactions in our previously published two-hit murine model system for PD may impact the microbiota. Notably, the lack of significant differences in diversity metrics and in compositional analyses between WT and *Pink1*^−/-^ mice at each time point during *C. rodentium* infection further serves as evidence that these mice are processing the infection similarly and have minimal intestinal disturbance. This suggests that other features of *C. rodentium* infection, such as immune activation, may be more directly involved in the development of the observed motor phenotypes. The role of SCFAs in *C. rodentium* infection, intestinal inflammation, and the gut-brain axis also need to be further elucidated. Although exogenous treatment with SCFAs has been noted to affect *C. rodentium* infection,^55^ to our knowledge, this is the first study to look at how *C. rodentium* affects the levels of various gut microbiota-derived SCFAs. The difference we observed in the levels of butyric acid between WT and *Pink1*^−/-^ mice may provide a possible source by which the intestinal milieu can alter immune responses in *Pink1*^−/-^ mice; however, more in-depth studies are needed to fully understand its role in PD and in various PD model systems.
